# The inter- and intra-rater reliability of the Maestro and Barroco metatarsal length measurement techniques

**DOI:** 10.1186/s13047-018-0289-7

**Published:** 2018-08-16

**Authors:** Zainab Ali, Hassan Karim, Navid Wali, Reza Naraghi

**Affiliations:** University of Western Australia, School of Surgery, Podiatric Medicine, Crawley, Western Australia 6009 Australia

**Keywords:** Metatarsal length, Reliability, Radiographic measurements, Maestro technique, Barroco technique

## Abstract

**Background:**

The relationship between metatarsal length and various forefoot pathologies is a topic of contention in Orthopaedics. The results of such investigations have been shown to depend on the method of metatarsal length measurement used. The aim of this study was to assess the inter- and intra-rater reliability of the Maestro and Barroco metatarsal length measurement techniques.

**Methods:**

A retrospective and quantitative study was performed on 15 randomly selected radiographs to determine the reliability of the two measurement techniques across all five metatarsals (M1 to M5). This was done at one week apart for three weeks by three raters. The intraclass correlation coefficient (ICC), and the 95% lower confidence limit (95% LCL) were calculated.

**Results:**

The Maestro and Barroco techniques produced high to very high ICC vlaues for length measurements across all metatarsals. The 95% lower confidence limit for inter-rater measurements ranged between 0.92–0.98 for Maestro’s and 0.86–0.99 for Barroco’s technique. For intra-rater measurements the 95% LCL ranged between 0.83–0.99 for Maestro’s and 0.75–0.99 for Barroco’s technique.

**Conclusions:**

Our study found that both the Maestro and Barroco methods of measurements produced high to very high inter- and intra-rater reliability. Both methods may be suitable for the use of peri-operative planning and clinical research relating metatarsal length and forefoot pathology. Besides having a more simplistic method of application, the novel Barroco technique is comparable to the more established Maestro method in both repeatability and reproducibility.

## Background

**Fig. 1:**
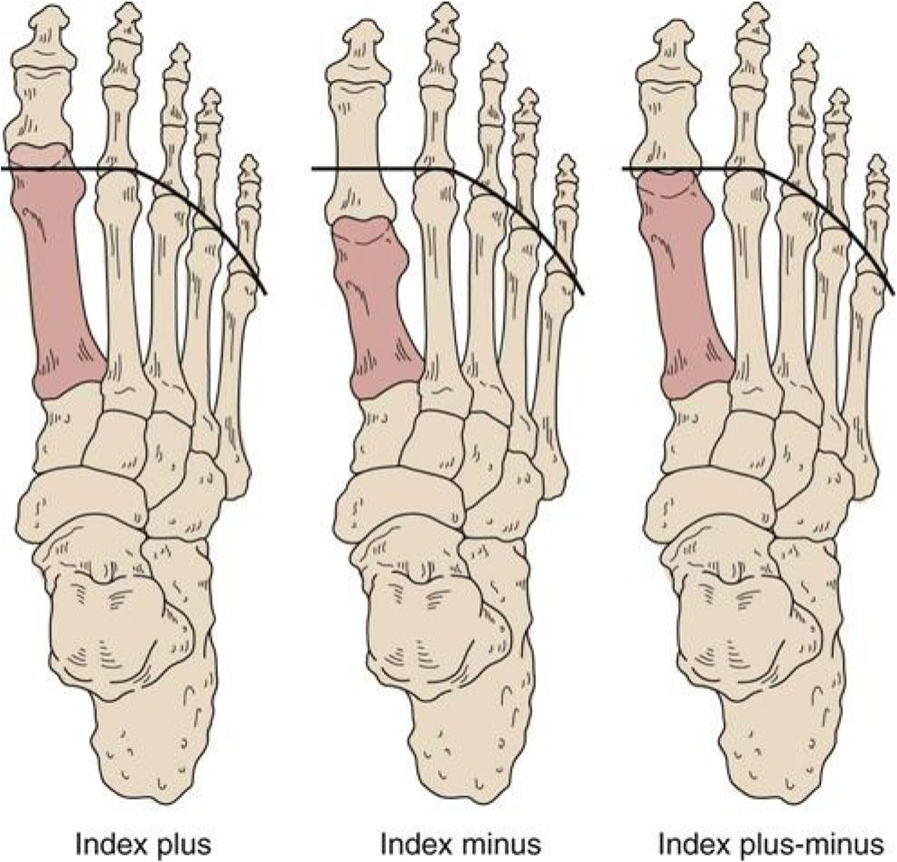
Forefoot morphotypes: Index-plus foot is also known as Egyptian foot. Index-minus foot is also known as Morton’s foot type or Greek foot. Index plus-minus foot is also known as the Roman foot

Metatarsal length has been an area of contention in Podiatric Medicine. Different forefoot morphologies due to variation in first metatarsal length relative to the 2nd metatarsal has led to multiple terms, such as Greek foot (index minus), Egyptian foot (index plus) and Roman foot (index plus-minus) as depicted in Fig. 1 [1]. These forefoot morphologies have been controversial in the literature, especially in relation to normality and possible association with forefoot pathology [1–9]. Metatarsal length and its association to hallux abductovalgus [10–15], Morton’s neuroma [16], forefoot plantar pressures [17–21] and metatarsophalangeal joint instability [22] have all been investigated.

However it is generally accepted that too long or too short metatarsal length can lead to forefoot pathologies [3, 23, 24]. Many forefoot corrective surgeries involve metatarsal shortening indicating a potential link between metatarsal length and forefoot pathologies [25–27]. A study by Pérez-Muñoz et al. tested the efficacy of Weil and triple Weil osteotomies for the treatment of metatarsalgia (*n* = 93 ft) [26]. Prior to surgery, majority of feet were classified as index-minus (*n* = 75). Post-operatively, the foot morphology was altered such that the majority were categorised as index plus-minus (*n* = 81). The authors noted good surgical results in 80% of the patients. Similar positive surgical outcomes were obtained by Devos Bevernage and Leemrijse study that used Maestro’s measurement tool for preoperative planning of Weil osteotomy [28]. Other studies have attained comparable findings in support of metatarsal shortening to relieve forefoot pain, even in regards to Morton’s neuroma [24, 27, 29]. However, there have been reports of surgical alterations in literature that have resulted in increased weight transfer to adjacent metatarsals post-operatively [30, 31]. This highlights the importance of a reliable radiographic measurement tool for peri-operative planning.

The method used in determining the extent of metatarsal shortening varies between surgeons and is widely undescribed [24, 32–34]. Davies and Saxby [34] proposed to shorten the lesser metatarsal until the tension on the surrounding soft tissue was released and the metatarsal-phalangeal joint was reduced. They would only shorten the second metatarsal up to 5 mm and would take extra care not to reduce it more than the third metatarsal in order to avoid transfer lesions. Some surgeons explicitly rely on Maestro’s idea of maintaining a “harmonious curve” to assure physiological function and correct weight distribution at the forefoot [32]. The harmonious forefoot morphotype is described as a geometrical progression of the relative lengths of the lesser metatarsals (eg. 1 ≤ 2 > 3 > 4 > 5) by a factor of two and deviations from this norm are considered to result in “disharmony” and hence result in a symptomatic forefoot [35]. As metatarsal shortening of as little as 2 mm can cause recurrence and transfer metatarsalgia [32], this further necessitates the use of a precise measurement technique in preoperative planning.

To date, there is no gold standard radiographic method for measuring metatarsal lengths [21] and there is a lack of agreement between different measurements methods [22, 23, 36]. Morton’s transverse lines’ [5], Coughlin’s [33], Maestro’s [35] and Hardy and Clapham’s [3, 10] methods are some of the commonly noted techniques in the literature [23]. Of these, Maestro’s technique is readily applicable to all five metatarsals along with a new un-validated method by Barroco et al. [2]. Our objective was to investigate the reliability and practicality of these two techniques and validate their use in future studies and peri-operative settings.

## Methods

**Fig. 2:**
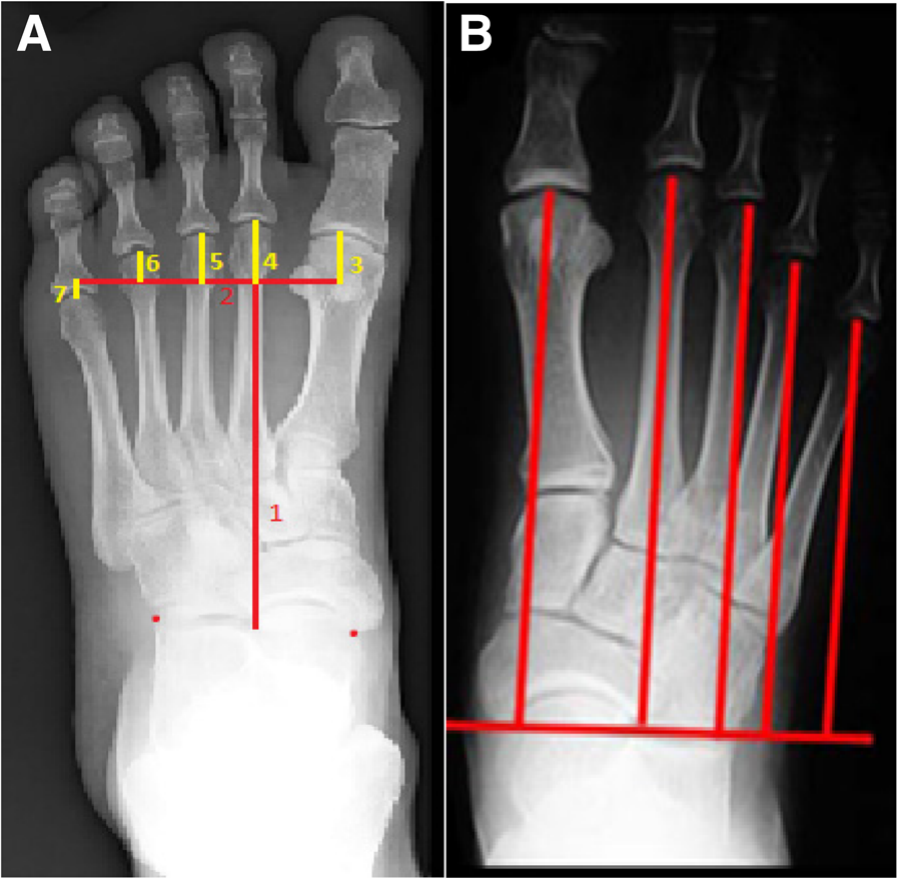
**a** Maestro’s technique involves seven lines; 1) Extends from the midpoint of the Chopart’s joint to the distal apex of the second metatarsal head, 2) Perpendicular line bisects the fibular sesamoid and extends across the metatarsal heads [SM4 line], 3–7) Vertical lines extending from the distal tips of metatarsals 1–5 to line 2. **b** Barroco’s technique involves six lines; Initially a proximal line is drawn extending between the most proximo-medial aspect of the navicular to the lateral congruence of the calcaneocuboid joint. A perpendicular line is then drawn from the apex of each metatarsal head to this proximal line

The aim of this study was to assess the inter- and intra-rater reliability of the Maestro (Fig. 2a) and Barroco (Fig. 2b) techniques used to measure metatarsal length radiographically. A retrospective and quantitative study was performed at the University of Western Australia (UWA) podiatry clinic. Ethics approval was obtained prior to the study.

Fifteen weight-bearing dorsoplantar (DP) radiographs from participants aged between 20 to 65 years were selected at random. All participants had signed informed consent allowing their radiographs to be used for future research by students of the UWA podiatry clinic. Participants were screened through the Genie Medical Software to exclude any remarkable forefoot deformities and surgical interventions, as highlighted by their medical history.

Three final year post-graduate podiatry students were initially trained by a specialist podiatric surgeon to conduct the radiographic measurements using the Digital Imaging and Communications in Medicine (DICOM) program, InteleViewer. Each of the three raters measured all five metatarsal lengths using both techniques within the same setting and time. Each rater conducted measurements in an isolated cubicle within the clinic. This was carried out at one-week intervals for three weeks. The digital weightbearing DP radiographs of the 15 participants were obtained through Perth radiology clinic, SKG and Imaging Central databases.

In order to determine the intra- and inter-rater reliability of the measurement techniques, intraclass correlation coefficients (ICCs) and the 95% lower confidence limit (95% LCL) were calculated for the lengths of metatarsal one (M1) to metatarsal five (M5). A two way mixed effect model with absolute agreement was utilized for calculating ICCs. The single measures ICCs were used as the measure of intra-rater reliability. The average measures ICCs were used as the measure of inter-rater reliability. The test was chosen to show if the measurements were in agreement within and between the raters. The reliability was regarded as minimal for ICC ≤ 0.25, low for ICC between 0.26 to 0.49, moderate for ICC between 0.50 to 0.69, high for ICC between 0.70 to 0.89 or very high for ICC ≥ 0.90 as originally used by Shima et al. [37]. According to a review article on determination of sample size requirements for estimating the value of intraclass correlation coefficient, for an ICC value above 0.80, total number of subjects needed were 6, with 3 measurements per subject to give the study 90% power [38]. Our study included 15 subjects with 3 measurement-repeats on each subject and depicted ICC values > 0.80. This allowed the study 90% power to reach valid conclusions on intra and inter-rater measurement reliabilities.

## Results

The mean and standard error of the mean (SEM) values in centimetre for each metatarsal length by each rater is given for Maestro and Barroco’s technique in Tables 1 and 2 respectively.

**Table 1:** Mean metatarsal length with SEM (in cm) for Maestro’s technique

Maestro measurements	Mean *N* = 45	SEM
Met_1_R1	1.218	.027
Met_1_R2	1.249	.025
Met_1_R3	1.206	.028
Met_2_R1	1.531	.039
Met_2_R2	1.554	.039
Met_2_R3	1.517	.039
Met_3_R1	1.095	.045
Met_3_R2	1.121	.047
Met_3_R3	1.076	.046
Met_4_R1	.251	.044
Met_4_R2	.286	.046
Met_4_R3	.239	.044
Met_5_R1	−1.145	.039
Met_5_R2	− 1.129	.040
Met_5_R3	−1.170	.041

Measurement data for each metatarsal by each rater was pooled from 15 subjects and 3 repeats per subject over three weeks, and mean and SEM calculated. *M* Metatarsal, *R* Rater

**Table 2:** Mean metatarsal length with SEM (in cm) for Barroco’s technique

Barroco measurements	Mean N = 45	SEM
Met_1_R1	12.251	.161
Met_1_R2	12.316	.156
Met_1_R3	12.304	.145
Met_2_R1	12.491	.181
Met_2_R2	12.364	.172
Met_2_R3	12.488	.169
Met_3_R1	11.997	.180
Met_3_R2	11.820	.166
Met_3_R3	11.979	.168
Met_4_R1	11.104	.171
Met_4_R2	10.855	.156
Met_4_R3	11.072	.160
Met_5_R1	9.668	.154
Met_5_R2	9.341	.143
Met_5_R3	9.608	.146

Measurement data for each metatarsal by each rater was pooled from 15 subjects and 3 repeats per subject over three weeks, and mean and SEM calculated. *M* Metatarsal, *R* Rater

### The inter-rater reliability

The inter-rater reliability results for the metatarsal length measurement for each week for Maestro’s and Barroco’s techniques are shown in Tables 3, 4 and 5. For Maestro’s technique, the 95% LCL of the ICC for measuring metatarsal length between raters exceeded 0.90 across all metatarsals, and the LCL values ranged 0.92 to 0.98. For Barroco’s technique, the 95% LCL was marginally below the 0.90 level for metatarsal five (M5) measurements in the first two weeks but surpassed the 0.90 level by week three. The LCL values for metatarsal length measurement using Barroco’s technique ranged between 0.86 to 0.99. The 95% lower confidence limit values indicate that both Barroco’s and Maestro’s measurement techniques produce high to very high reliability in measuring length across all metatarsals.

**Table 3:** Intraclass correlation coefficients and 95% lower confidence limit on measurements of raters from week one

Metatarsals	Barroco		Maestro	
	ICC	LCL	ICC	LCL
M1	0.990	0.977	0.983	0.960
M2	0.988	0.970	0.987	0.969
M3	0.984	0.962	0.994	0.986
M4	0.971	0.927	0.991	0.976
M5	0.946	0.865	0.991	0.978

Two-way mixed effects model used where people effects are random and measures effects are fixed. Inter-rater ICCs are obtained using metatarsal 1–5 length measurements from all raters in week 1, where *n* = 15

**Table 4:** Intraclass correlation coefficients and 95% lower confidence limit on measurements of raters from week two

Metatarsals	Barroco		Maestro	
	ICC	LCL	ICC	LCL
M1	0.987	0.969	0.982	0.944
M2	0.992	0.981	0.994	0.986
M3	0.988	0.971	0.991	0.979
M4	0.976	0.934	0.967	0.921
M5	0.954	0.871	0.980	0.953

Two-way mixed effects model used where people effects are random and measures effects are fixed. Inter-rater ICCs are obtained using metatarsal 1–5 length measurements from all raters in week 2, where *N* = 15

**Table 5:** Intraclass correlation coefficients and 95% lower confidence limit on measurements of raters from week three

Metatarsals	Barroco		Maestro	
	ICC	LCL	ICC	LCL
M1	0.993	0.983	0.983	0.947
M2	0.996	0.991	0.992	0.977
M3	0.995	0.986	0.992	0.953
M4	0.993	0.978	0.974	0.938
M5	0.988	0.959	0.991	0.970

Two-way mixed effects model used where people effects are random and measures effects are fixed, Inter-rater ICCs are obtained using metatarsal 1–5 length measurements from all raters in week 3, where *N* = 15

### The intra-rater reliability

The intra-rater reliability is presented in Tables 6 and 7 for Maestro’s and Barroco’s measurement techniques respectively using three repeats of the measurement by each rater. Considering the 95% lower confidence limit, the intraclass correlation coefficients for measurement of metatarsal lengths (M1 to M3) exceeded the 0.90 level for both measurement techniques. However for some raters, LCL was below the 0.90 level for metatarsal four (M4) measurement using Maestro’s technique and M4 & M5 measurement using Barroco’s techniques. Where 95% lower confidence limit ICC for M4 for both techniques were generally above 0.80 level; LCL for M5 ranged between 0.75–0.96 using Barroco’s technique and 0.90–0.98 using Maestro’s technique. Maestro technique showed a tendency to produce lower intra-rater variability in measuring M5 over Barroco’s technique.

**Table 6:** Intraclass correlation coefficients and 95% lower confidence limit within rater measurements for Maestro’s measurement technique

Metatarsals	Rater 1		Rater 2	Rater 3		
	ICC	LCL	ICC	LCL	ICC	LCL
M1	0.993	0.982	0.973	0.939	0.969	0.929
M2	0.994	0.998	0.978	0.949	0.992	0.980
M3	0.996	0.990	0.985	0.949	0.988	0.973
M4	0.992	0.982	0.934	0.854	0.923	0.832
M5	0.993	0.984	0.987	0.969	0.960	0.909

Two-way mixed effects model used where people effects are random and measures effects are fixed. Intra-rater ICC obtained on three repeats of measurements by each rater with *N* = 15

**Table 7:** Intraclass correlation coefficients and 95% lower confidence limit within rater measurements for Barroco’s measurement technique

Metatarsals	Rater 1		Rater 2		Rater 3	
	ICC	LCL	ICC	LCL	ICC	LCL
M1	0.987	0.969	0.966	0.923	0.986	0.968
M2	0.997	0.993	0.985	0.965	0.988	0.971
M3	0.992	0.982	0.969	0.930	0.983	0.960
M4	0.993	0.983	0.937	0.861	0.974	0.940
M5	0.987	0.969	0.883	0.752	0.953	0.895

Two-way mixed effects model used where people effects are random and measures effects are fixed. Intra-rater ICC obtained on three repeats of measurements by each rater with *N* = 15

## Discussion

The reproducibility (inter-rater reliability) of a test indicates the precision of a method and determines its validity and use in clinical practice [39]. The repeatability (intra-rater reliability) refers to the variation in repeat measurements by the same rater under identical conditions. The results from the present study showed that both the Maestro and Barroco methods depicted excellent levels of reproducibility and repeatability.

Maestro’s method of measurement depicted inter-rater LCL values ranging from 0.921–0.986 across all five metatarsals, over the three weeks. Our study implies that the Maestro technique has very high reliability for all five metatarsals between raters. The intra-rater LCL values ranged from 0.832–0.998 across all five metatarsals for the three raters. Overall, our results are in concordance with Maestro et al.’s original paper, which reported “excellent” reliability outcomes [35]. However, they failed to provide information on how they reached these conclusions [35]. Our results are further supported by Deleu et al. who found inter-rater ICC values ranging from 0.982–0.997 and intra-rater ICC results between 0.981–0.997 [32]. It is worth noting both our study and Deleu et al. used Maestro’s technique for metatarsal length measurement. However, their ICC values were based on the agreement of two observers in regards to forefoot morphotype classification, and hence they fail to provide length measurements in their study [32]. In contrast, when testing for inter-rater and intra-rater variability using the 95% limits of agreement, Chauhan et al. found “high variability” between and within raters using the Maestro technique [36]. This may be due to the time-gap between measurements; while their study collected measurements three months apart, we conducted ours weekly. A comparison of relative metatarsal length in normal feet between our study and Maestro’s study is given in Table 8 below. The comparability of metatarsal lengths between the two studies further validates maestro’s measurement technique.

**Table 8:** Comparison of mean relative metatarsal length ± SEM (in mm) in normal feet between Maestro’s study and our study

	Mean ± SEM (in mm)			
	M2-M1	M2-M3	M3-M4	M4-M5
Maestro et al. [43] *N* = 40	3.3 ± 0.9	3.3 ± 0.9	6.5 ± 1.0	12 ± 1.9
Our study N = 15	3.1 ± 0.3	4.3 ± 0.4	8.5 ± 0.4	13.9 ± 0.4

Barroco’s method of measurement showed inter-rater LCL values between 0.865–0.998 across all five metatarsals. The intra-rater LCL values were found to be between 0.752–0.993 across all five metatarsals. As we are the first study to investigate the reliability of this measurement technique, there is no relevant literature to support or refute our reliability findings. However the absolute metatarsal lengths in normal feet were comparable between our study and the original Barroco study (Table 9). In both studies index minus foot type was most prevalent with metatarsal formula 1 < 2 > 3 > 4 > 5. The observed metatarsal length variability between the two studies could relate to differences in sample size and gender disparity in the study population. Where Barroco et al. studied metatarsal length in 83 male and 83 female normal feet (*n* = 332 ft), our study examined metatarsal length on a total of 15 normal feet from a pooled sample of male and female radiographs. It is well established that on average male foot is inherently longer than that of a female [40, 41].

**Table 9:** Comparison of mean relative metatarsal length and standard deviation (SD) in millimetres in normal feet between Barroco and our study

	Mean (SD) (in mm)									
	M1		M2		M3		M4		M5	
	Male	Female	Male	Female	Male	Female	Male	Female	Male	Female
Barroco et al. [2]	125.4	115.1	127.8	117.3	123.4	113.5	114.2	105.3	99.5	91.7
*N* = 83 male										
*N* = 83 female	(8.2)	(7.4)	(8.2)	(7.3)	(8.0)	(7.2)	(7.7)	(7.2)	(7.9)	(7.4)
Our study *N = 15	122.8 (10)		124.2(11)		119.0 (11)		109.0 (10)		94.6 (9)	

*N = 15 includes male and female subjects

One limitation of our study was that we couldn’t control for any variability stemming from imaging protocol. During the initial investigation of both techniques, we noticed that the angulation of the proximal reference line seemed to depend on the rear-foot positioning (Fig. 2) as also mentioned by Deleu et al. [32]. For example, a significantly pronated versus supinated foot may have influenced the angulation of the proximal reference line [32]. Future studies can standardise the rear-foot positioning by taking the weight-bearing DP radiographs in neutral calcaneal stance position (NCSP). Furthermore prospective studies could consider standardising x-ray imaging conditions (e.g. X-ray source inclination of 15° with beam centred between the navicular bones, distance from the foot to the X ray source = 1 m) as also suggested by previous studies to minimise sources of variability [2, 35].

Though we excluded any forefoot pathology in our study sample, presence of forefoot deformity such as hallux valgus is likely to change the SM4 reference line by changing the fibular sesamoid position in Maestro’s technique. But because the measurement is the measurement of relative lengths or distances, this doesn’t change the reliability of the measurement technique. In fact Maestro has reported excellent intra-observer and inter-observer reproducibility in metatarsal length measurement in feet with hallux valgus and rigidus [35].

The practicality of both methods should not be overlooked. The Barroco technique requires only one line to be drawn between easily recognisable points before making metatarsal length measurements. It is simple, easy to use on any foot morphotypes, to little variation and does not require complex instruments. The Maestro technique requires several steps, each dependent on the other. This may be more time consuming and requires proper training to conduct measurements.

We believe that the use of both Maestro and Barroco methods may help clinicians in the peri-operate planning relating to forefoot procedures. The subsequent biomechanical implications following shortening osteotomies and general forefoot procedures are not well understood at this stage [24, 25, 42]. The use of objective metatarsal length measurements peri-operatively using the Maestro and/or Barroco techniques can lead to better understanding in this field.

## Conclusion

This study shows that both the Maestro and Barroco metatarsal length measurement techniques produce high to very high repeatability and reproducibility across all five metatarsals. We deem both methods reliable for the purpose of forefoot procedures peri-operative planning and research investigating metatarsal length and forefoot pathology. We found both methods very practical to conduct. The novel Barroco method was more simplistic. Our study supports the use of this method for future use.

## Abbreviations

DICOM: Digital imaging and communications in medicine
DP: Dorsoplantar
ICC: Intraclass correlation coefficient
LCL: Lower-confidence limits
M1-M5: Metatarsal one to metatarsal five
